# Artificial intelligence and echocardiography

**DOI:** 10.1530/ERP-18-0056

**Published:** 2018-10-29

**Authors:** M Alsharqi, W J Woodward, J A Mumith, D C Markham, R Upton, P Leeson

**Affiliations:** 1 Oxford Cardiovascular Clinical Research Facility, Division of Cardiovascular Medicine, Radcliffe Department of Medicine, University of Oxford, Oxford, UK; 2 Ultromics Ltd, Magdalen Centre, Robert Robinson Ave, Oxford, United Kingdom

**Keywords:** echocardiography, artificial intelligence, machine learning

## Abstract

Echocardiography plays a crucial role in the diagnosis and management of cardiovascular disease. However, interpretation remains largely reliant on the subjective expertise of the operator. As a result inter-operator variability and experience can lead to incorrect diagnoses. Artificial intelligence (AI) technologies provide new possibilities for echocardiography to generate accurate, consistent and automated interpretation of echocardiograms, thus potentially reducing the risk of human error. In this review, we discuss a subfield of AI relevant to image interpretation, called machine learning, and its potential to enhance the diagnostic performance of echocardiography. We discuss recent applications of these methods and future directions for AI-assisted interpretation of echocardiograms. The research suggests it is feasible to apply machine learning models to provide rapid, highly accurate and consistent assessment of echocardiograms, comparable to clinicians. These algorithms are capable of accurately quantifying a wide range of features, such as the severity of valvular heart disease or the ischaemic burden in patients with coronary artery disease. However, the applications and their use are still in their infancy within the field of echocardiography. Research to refine methods and validate their use for automation, quantification and diagnosis are in progress. Widespread adoption of robust AI tools in clinical echocardiography practice should follow and have the potential to deliver significant benefits for patient outcome.

## Background

Echocardiography plays a crucial role in the diagnosis and management of patients with cardiovascular disease. Since echocardiography is the only imaging modality that permits real-time imaging of the heart, it allows the immediate detection of various abnormalities (1). The accurate quantitative assessment of cardiac structure and function is essential for clinical diagnosis and to help guide the most appropriate treatments. However, despite the abundance of guidelines for the interpretation and assessment of echocardiograms, quantification and diagnosis based on subjective review of 2D echocardiography remains imperfect and prone to error (2). It is a long-standing issue that there is a reasonably high level of inter-observer variation in the interpretation of echocardiograms, especially amongst those with poor-quality images (3).

Although AI has been around since the 1950s, there has only recently been a surge of interest and research in the use of AI in medical imaging. AI techniques, such as machine learning, can be used to recognise a wide range of patterns within echocardiograms as they can take account of each pixel, and their relationship, as well as associated clinical metadata. Machine learning models can be trained to ‘learn’ what different features in an image represent so that they can be used to identify images, quantify areas of interest or be associated with particular disease patterns (4).

By combining clinician interpretation with information derived from machine learning algorithms, there is the opportunity to enhance the accuracy of echocardiography through a reduction in inter- and intra-operator variability, as well as providing additional predictive information that may be too subtle to be detected by the human eye (5, 6, 7). As such, machine learning models show promise as tools for the rapid, accurate and precise assessment of cardiovascular structure and function, which could pave the way for a new era of echocardiography. In this review we consider what artificial intelligence is, some of the reasons why it is particularly applicable to echocardiography and provide examples of the current state-of-the-art applications.

## What is artificial intelligence?

AI is defined as the ability of computer systems to perform tasks that would usually require human levels of intelligence. A subfield of AI is machine learning which can be used to teach a computer to analyse a vast number of data points in a rapid, accurate and efficient manner through the use of complex computing and statistical algorithms. These algorithms infer relationships from existing datasets and learn which of these relationships have the highest predictive power. By harnessing this knowledge, machine learning models are then able to make predictions based on unseen data (8).

**Figure 1:**
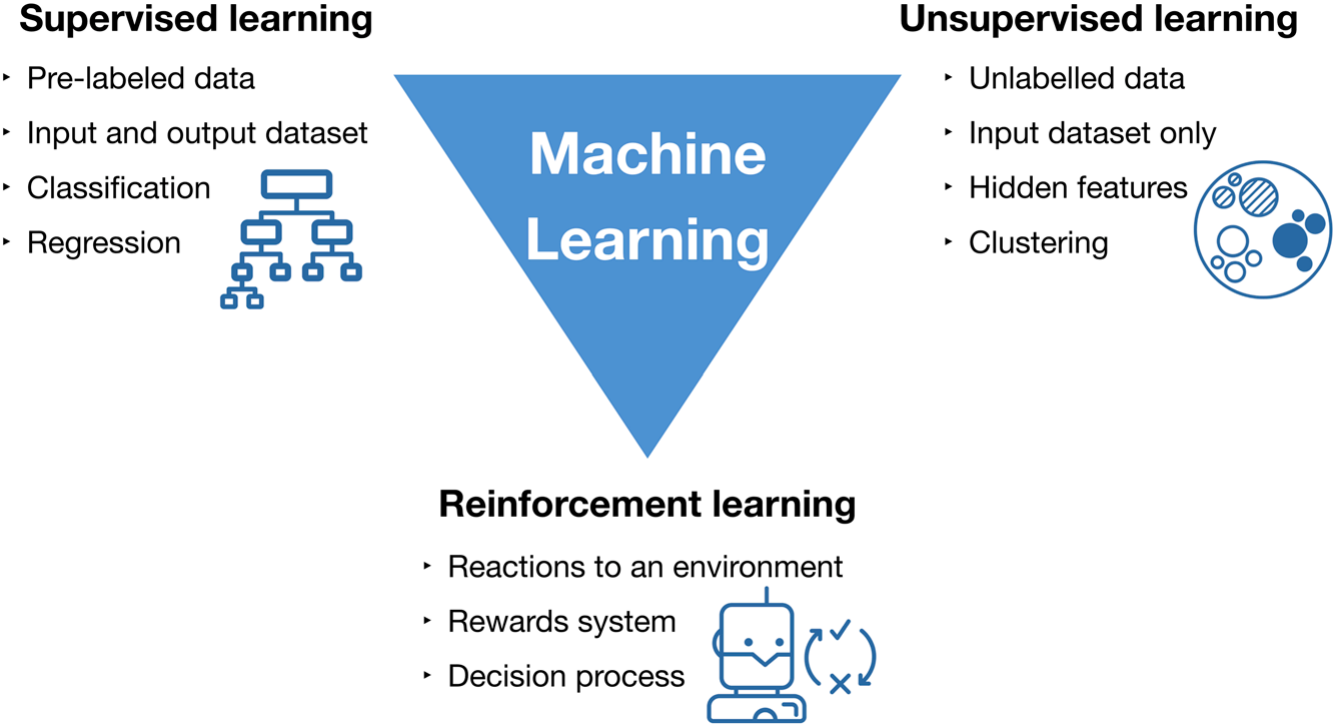
Types of machine learning algorithms.

Machine learning can be classified into three groups supervised, unsupervised and reinforcement learning (Fig. 1 and Table 1) (9). In supervised learning, the machine is ‘taught’ to classify data by providing it with a training dataset of labelled data. During the training process, the machine learning algorithm learns underlying patterns within the data and compares these with known outcomes. Once the machine has been trained, its ability is tested using an unseen dataset. This allows the assessment of the accuracy of the model and how it compares to human interpretation (4, 10). Examples of supervised learning methods include random forests, support vector machines and artificial neural networks. The current artificial neural networks are only a few sets of neuron layers so, at best, represent the outermost layer of cortex and not the full brain. In contrast, unsupervised learning techniques uses unlabelled data to classify the input data into multiple groups, or clusters, based on similarities between the data points (10, 11). Reinforcement learning is based on interactions with an environment. The learner, also called the agent, attempts to discover the most successful actions to achieve maximum reward by learning from trial and error. This leads to an agent suitable for dynamic environments (12, 13). It should be noted however that these methods of machine learning are not mutually exclusive. Deep learning, a type of artificial neural network capable of handling larger, more complex datasets using techniques such as convolutional neural networks, can be either supervised, unsupervised or semi-supervised (4). Also, the subfields within machine learning have been integrated together to create even more powerful techniques, such as deep reinforcement learning (14, 15).

**Table 1:** Definition of machine learning classes.

Class of machine learning	Definition
**Supervised learning**	Uses human-coded information to train machine learning models to classify unseen data.
Random forest	This is an ensemble of decision trees. An item is classified according to the most common output from all of the decision trees. Due to the increased exposure to samples of training data, random forests have the advantage of not over-fitting a model to the data, compared to a single decision tree.
Support vector machines (SVMs)	SVMs allow the construction of models capable of separating training data into different classes. When presented with new data, these models are able to predict which class it should belong to.
Artificial neural networks (ANNs)	ANNs are modelled on the design of the brain. Due to their structure of interconnecting layers of neurons, artificial neural networks have been likened to the outer cortex of the brain. These networks are comprised of interconnected layers that are involved in the analysis and classification of input data. The greater the number of layers a network has, the higher the level of analysis; this forms the basis for deep learning. ANNs are able to learn which connections are the most useful for classifying data and thus weight these accordingly.
**Unsupervised learning**	The model is not provided with human-coded outcomes, so the model has to classify data itself based on its own analysis. This has the potential to identify novel relationships within the data.
Clustering techniques	This method is similar to SVMs, however as the data is unlabeled, the model is unable to classify it based on human-coded information. Instead, the model identifies the natural groupings, or clusters, of data and uses these clusters to classify new data.
Naive Bayes	These are a family of techniques which apply Bayes’ Theorem (Bayes’ Theorem states that the probability of an event can be affected by prior evidence) to classify data, assuming that the data are independent from each other.
Principal component analysis (PCA)	PCA is a technique that makes data easier to analyse by transforming potentially correlated variables into non-correlated variables, known as principal components. These principal components allow for feature extraction from the original dataset.
Autoencoders	These are a type of ANN that encode the input into a compressed dataset, learn from this compressed information, and then reconstruct this information as output. By compressing the input data, this technique aims to learn the most important features of the input data.
**Reinforcement learning**	The machine learns how to interact with its environment through trial and error, to maximize the rewards. It is analogous to how a baby learns to interact with its environment.
Q-Learning	In Q-Learning, the agent learns how to optimally process different types of data in different ways.(NB. whilst this is a powerful machine learning technique, its use in the medical field is limited at present).

Model training is a process common to all types of machine learning. It is the process whereby the model analyses a variety of features within the data provided and uses these to learn how to generate an output label. For example, within echocardiography, a model could analyse a variety of features such as left ventricular wall thickness and left ventricular ejection fraction to determine whether a patient has a particular condition. However, the inclusion of irrelevant features in the analysis can lead to over-fitting of the model, thus rendering it less accurate when presented with new datasets. This emphasises the importance of having a training dataset that is representative of the population. Information gain analysis is used to determine which features to include in the model to ensure that it has the highest predictive power, yet can still be applied to other populations (10, 16). Once the model is trained, it can be applied to unseen datasets to test its predictive ability before being used fully.

Whilst machine learning models provide the potential to be able to rapidly analyse large volumes of data, they, themselves, require large volumes of data to ensure that they are thoroughly trained. In the medical field, access to this data can be difficult to obtain and, once obtained, requires a great deal of effort to ensure that the data is clean, of sufficient quality and accurate before being used to train the model. Furthermore, it is important to ensure that these datasets are representative of the population the model will be used for, since sampling bias and missing data can negatively affect the predictive ability of the model. Data leakage, the unintentional use of training data to test the model’s accuracy, can lead to an inaccurate assessment of a model’s predictive power (17), this demonstrates the need for a thorough training process.

Whilst it is anticipated that these models will provide additional information and act as a tool to guide clinicians in the decision making process, it is important for clinicians to bear the limitations of machine learning models in mind when employing them in clinical practice and to integrate their output into the wider clinical picture. Whilst these models have been designed to mimic human intelligence, their role is to identify correlations within data and classify it based on these correlations it does not provide any causal information. In addition, deep learning models are complex so that it can be difficult to understand how the model came to a particular conclusion (18). As such, it is up to the clinicians to identify the cause and decide on the most appropriate course of action.

AI, in particular machine learning, has been applied to a variety of techniques in the medical imaging field, for example calcium scoring in CT coronary angiograms, detection of diabetic retinopathy in retinal photography and skin cancer classification in dermatology (19, 20, 21).

## The need for AI in echocardiography

Echocardiography is an essential tool in the diagnosis and management of a wide range of cardiovascular diseases. As a result guidelines have developed to ensure accurate quantification and interpretation (22) but the final analysis remains reliant on the operator having the experience and knowledge to adhere to these guidelines. It may be possible to overcome or reduce this limitation by use of machine learning models. For example, guidelines recommend quantitative measures of chambers and valves during assessment to inform clinical decision-making (2, 23). However, in busy clinical environments such as acute emergency settings, quantitative analysis may not be practical because of the additional time required for manual tracing. Therefore, it is acknowledged that visual estimation remains the mainstay in many areas of clinical practice; although this requires considerable experience in echocardiography (24, 25). Application of machine learning to either highlight need for quantification or provide fully automated measures rapidly to the clinician could therefore overcome this issue and improve accuracy of diagnosis (26).

In busy clinical settings, there is also usually a narrow window in which to fully analyse and report the results of the scan and, as such, not enough time to analyse complex datasets (27). During a routine echocardiogram, a large volume of potentially diagnostically useful data are generated and with the advent of multi-dimensional imaging modalities, such as 3D echocardiography and speckle tracking, the volume of the data acquired is expected to increase (28, 29). Most data obtained remains under-utilised (5, 30) but machine learning techniques offer the potential to interpret simultaneously multiple datasets extracted from echocardiograms in an efficient and automated manner (10). Furthermore, these models are able to link available clinical data from electronic health records with echocardiography data, thus providing clinicians with more information, and allowing them to make more informed decisions about their care of patients (11).

One of the main advantages of using machine learning models within the interpretation process is that models can also bring these data together to act as predictive tools with potentially high levels of accuracy. Following training, the machine learning algorithm should be able to recognise different cardiac structural and functional patterns that, if subtle, could potentially be missed during the interpretation by the clinician. These data are predicted by comparing features from new data to a model fitted on features extracted from the training data (31, 32, 33).

**Figure 2:**
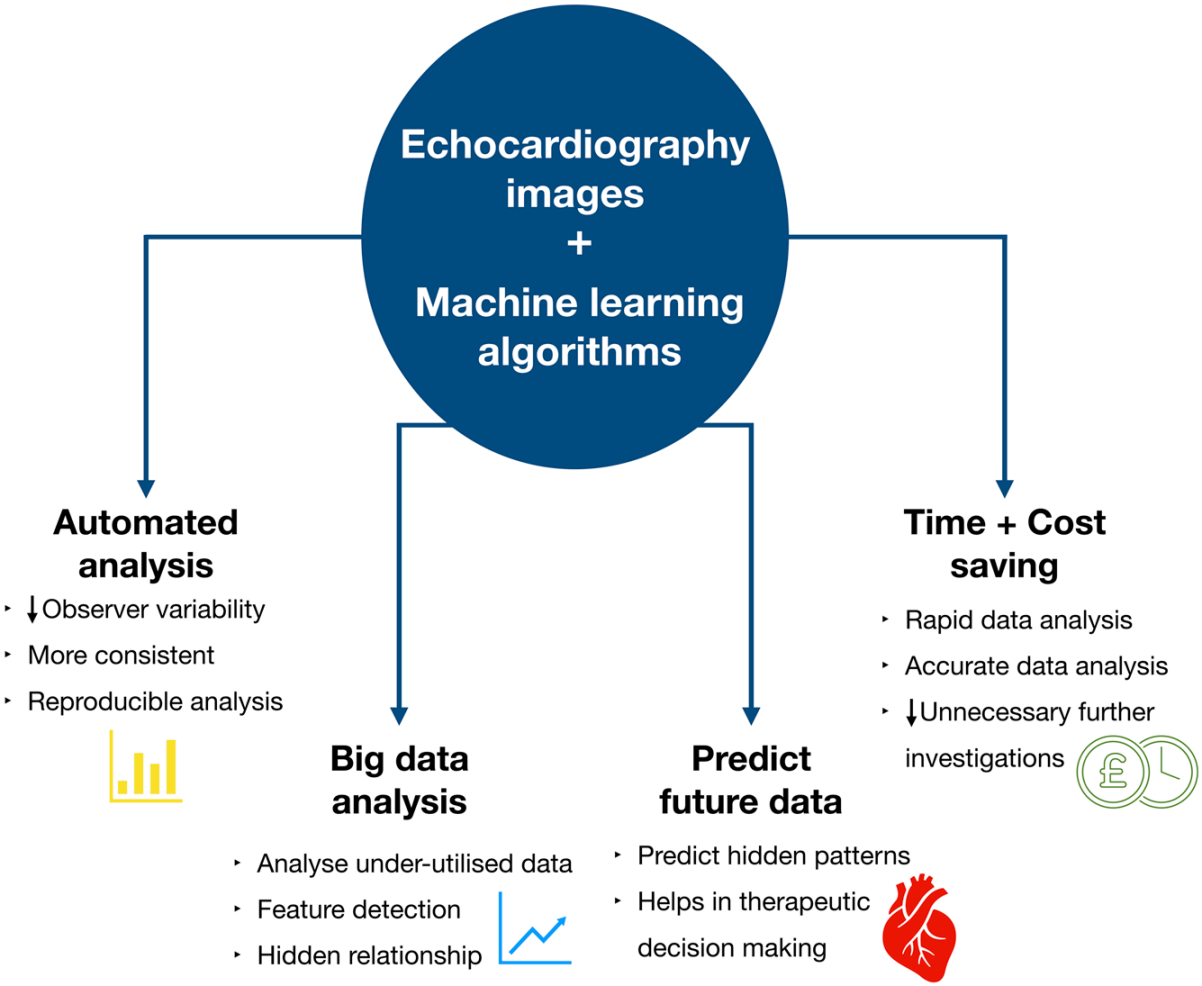
Advantages of machine learning assisted echocardiography interpretation.

Figure 2 provides a summary of these potential applications of machine learning within the field of echocardiography, which should not only make the interpretation process more accurate and reproducible, but also allow the incorporation of currently unused data into the overall assessment of cardiac function to provide a more accurate diagnosis. This increase in accuracy, combined with the time-saving benefits, suggest a real potential benefit for incorporation of machine learning into routine clinical echocardiography.

Machine learning models have also been shown to provide an almost instantaneous assessment of an echocardiogram. Knackstedt *et al.* demonstrated that left ventricular ejection fraction and longitudinal strain could be analysed in approximately 8 s (24). This rapid assessment of echocardiograms offers the potential to save the time of clinicians, who would otherwise have to manually derive these measurements and generate a report of their findings. Since the number of echocardiograms performed worldwide is increasing, this technology could allow for an increase in scanning, without the concomitant increase in reporting time that would otherwise limit their ability to see other patients. This rapid and accurate assessment could also have benefits extending beyond the Cardiology setting, to the Emergency Department where point-of-care ultrasound scans are becoming increasingly common. However, in this setting the operators are usually less experienced in image acquisition and interpretation (34). By incorporating machine learning algorithms, there is the potential to improve the diagnostic accuracy of echocardiography in the acute setting.

Machine learning has been demonstrated to provide numerous benefits to the field of echocardiography, which will not only make the interpretation process more accurate and reproducible, but will also allow the incorporation of currently unused data into the overall assessment of cardiac function to provide a more accurate diagnosis (Fig. 2). This increase in accuracy, combined with the time-saving benefits, demonstrate the value of incorporating machine learning into the field of echocardiography.

However, in order to incorporate this new technology into the healthcare setting the necessary infrastructure, such as computers, networks and servers capable of processing and securely transporting and storing such data, needs to be in place especially in the era of cloud-based systems. This may be a potential ‘bottle neck’ in the adoption of this technology, since healthcare IT systems are often in need of update (35). In addition, clinicians will need to be trained to ensure that these tools are used appropriately in order to get the most accurate information from the models, this is especially important as the validity of the output data is heavily dependent on the quality of the data fed into the model. As such, clinicians will have to work closely with data scientists to help facilitate the integration and adoption of these computational tools into the clinical setting (35).

## State of the art – future and potential applications

Although application of machine learning to echocardiography is at a relatively early stage, several applications have already been developed to facilitate interpretation. These methods cover image recognition, classification of pathological patterns and automated quantification. Table 2 summarises the accuracy, sensitivity and specificity of current machine learning applications in the field of echocardiography.

**Table 2:** Basic finding and validation of machine learning applications in the field of echocardiography.

Study	Year	Application	Machine learning model used	Training/validation set	Test set	Limit of agreements and bias	Sensitivity/Specificity/Accuracy	AUC	Time required for measurement
(7)	2018	Recognise 15 echocardiography views	Convolutional neural network	200,000 images	20,000 images	–	–/–/91.7%	0.996	21 ms/image
(54)	2018	Quantification of wall motion abnormalities	Double density-dual tree discrete wavelet transform	279 images	–	–	96.12%/96%/96.05%	–	–
(55)	2018	Quantification of wall motion abnormalities	Convolutional neural network	4392 maps	61 subjects		81.1%/65.4%/75%	–	–
(36)	2017	Recognition/classification of apical views	Supervised dictionary learning	210 clips	99 clips	–	–/–/95%	–	0.05 ± 0.003 s per clip
(57)	2017	Assessment of myocardial velocity	Unsupervised multiple kernel learning	55 subjects	–	Avg 51.7%	Avg 73.25%/78.4%/–	–	<30 s
(5)	2016	Classification/discrimination of pathological patterns (HCM vs ATH)	Support vector machine, random forest, artificial neural network	–	–	–	96%/77%/–	0.795	8 s
(27)	2016	Classification/discrimination of pathological patterns (RCM vs CP)	Associative memory-based machine-learning algorithm	–	–	–	–/–/93.7%	0.962	–
(47)	2016	Quantification of MR	Support vector machine	5004 frames	–	–	99.38%/99.63%/99.45%	–	–
(24)	2015	Calculation of EF and LS	AutoEF Software	–	255 patients	0.83 (0.78 to 0.86) and −0.3 (1.5 to 0.9)	–	–	8 ± 1 s/patient
(37)	2013	Automated detection of LV border	Random forest classifier with an active shape model	50 images	35 images	–	–/–/90.09%	–	–
(53)	2011	Quantification of wall motion abnormalities	Relevance Vector Machine classifier	173 patients	–	–	–/–/93.02%	–	–
(56)	2008	Quantification of wall motion abnormalities	Hidden Markov model	24 studies (720 frames)	20 studies (600 frames)	–	–/–/84.17%	–	–
(39)	2008	Calculation of EF	AutoEF Software	10,000 images	92 patients	1% (−19% to 33%)	–	–	–
(38)	2007	Calculation of EF	AutoEF Software	>10,000 images	200 patients	6% (−2.87 to 2.91)	–	–	<15 s per view

ATH, athletes’ heart; Avg, average; CP, constrictive pericarditis; EF, ejection fraction; HCM, hypertrophic cardiomyopathy; LS, longitudinal strain; LV, left ventricle; MR, mitral regurgitation; ms, milliseconds; RCA, restrictive cardiomyopathy; s, seconds.

### Image recognition

**Figure 3:**
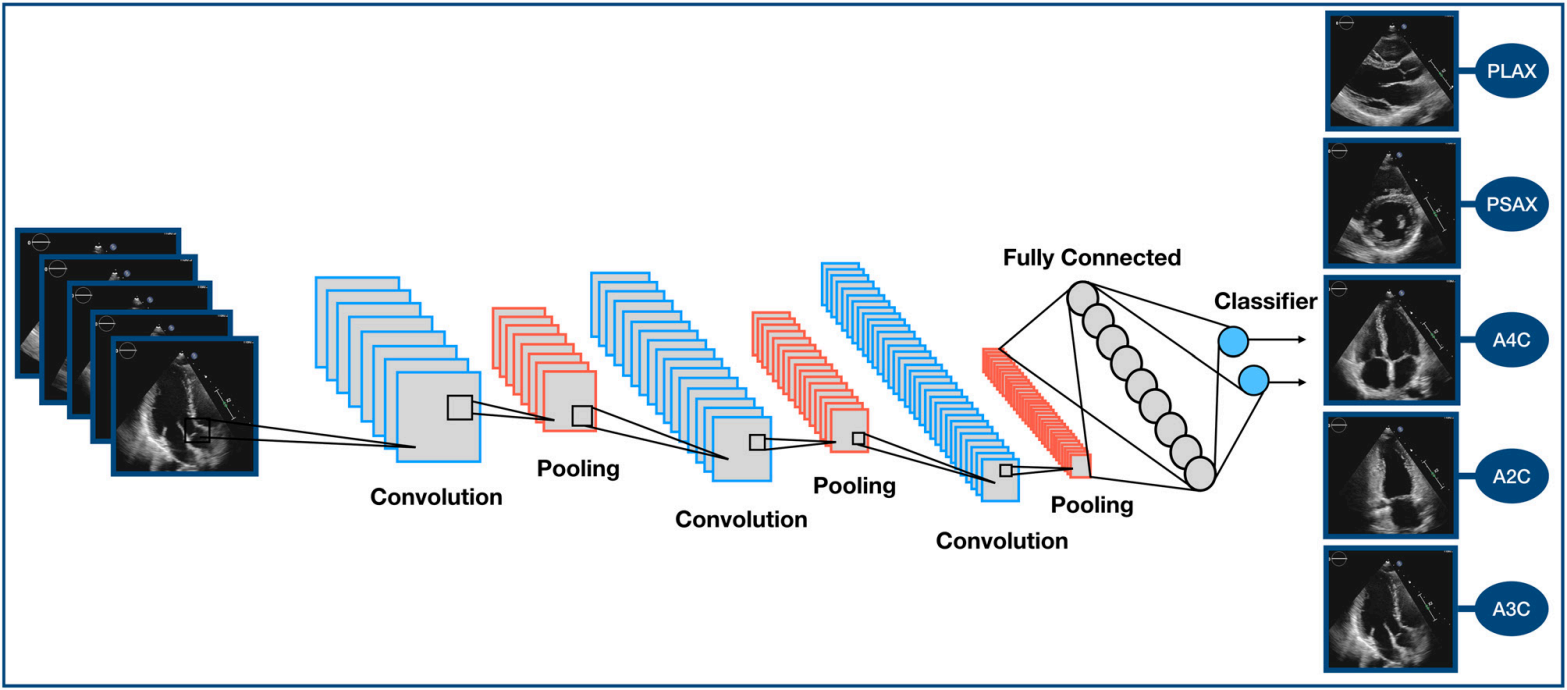
An example of a Convolutional Neural Network model for image classification. A2C, apical two chamber; A3C, apical three chamber; A4C, apical four chamber; PLAX, parasternal long axis; PSAX, parasternal short axis.

A first step to ensure the accurate assessment of echocardiograms is correct identification of views, videos and ultrasound modality such as pulsed and continuous wave Doppler traces. This recognition has proved relatively straightforward for machine learning applications. A deep learning model, consisting of a convolutional neural network, has been trained to detect and recognise specific features in each view, regardless of image resolution. In the study, a wide range of randomly selected echocardiograms, including normal variants, as well as a range of pathology and image qualities were used. The model was able to classify 15 major echocardiography views with an overall accuracy of 97.8% (7). In a separate study, machine learning algorithms were used to accurately identify apical four-, two- and three-chamber views. Despite the similarities between these views, the supervised learning model was able to recognise each view with an accuracy of approximately 95% (36). Figure 3 shows an example of a convolutional neural network model for echocardiography image classification.

### Classification of pathological patterns

**Figure 4:**
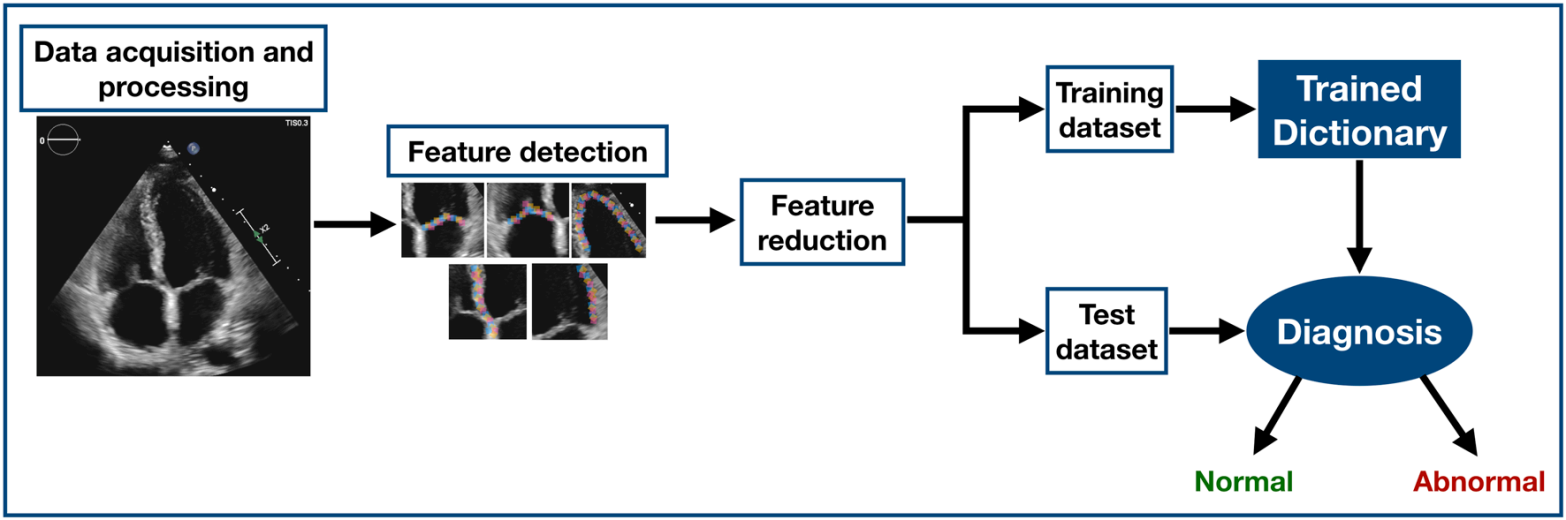
Diagram of an example of machine learning model process.

Different physiological and pathological conditions can share similar phenotypes that prove difficult to differentiate without detailed operator experience. For example, left ventricular hypertrophy is commonly observed in the athletic population, but is also found in hypertrophic cardiomyopathy. Given the increased risk of sudden cardiac death in inherited cardiac disease accurate differentiation is important. Narula and co-workers developed an ensemble technique consisting of support vector machines, random forests, and artificial neural networks to accurately differentiate between these two conditions, with a sensitivity of 96%, when adjusted for age (5). In addition to this, machine learning models have further demonstrated their ability to accurately differentiate between similar phenotypes in patient groups with constrictive pericarditis and restrictive cardiomyopathy. These pathologies share a similar presentation and since there is no single parameter in the field of echocardiography that can clearly distinguish between them, this diagnosis can be challenging. A machine learning classification algorithm was able to accurately differentiate between these pathologies, with accuracy of up to 90%, using multiple echocardiography features (27, 30). A diagram of an example of machine learning model process is shown in Fig. 4.

### Automated quantification

Echocardiography interpretation and guidelines rely heavily on use of quantitative measures. Image processing techniques with underlying machine learning algorithms have shown promise for rapid identification of structures and quantification of related parameters. Assessment of left ventricular volume and function was one of the first applications of artificial intelligence to minimise error and reduce operator subjectivity (37, 38, 39, 40). Methods have evolved so that, recently, Knackstedt *et al.* demonstrated that left ventricular ejection fraction and longitudinal strain could be analysed in approximately 8 s using machine learning methods (24). Within 3D echocardiography, random forest models to identify borders have been shown to provide an accurate identification of left and right ventricular cavities so that derived left and right ventricular volumes are comparable to those measured by cardiac magnetic resonance (28, 41, 42, 43, 44, 45). Furthermore, machine learning has been shown to aid in the assessment of valvular heart disease, for example, mitral valve disease (22, 46). Automated assessments of 3D transoesophageal echocardiograms of the mitral valve provided more reproducible and consistent quantitative assessment of the mitral valve annulus size and its morphology than human interpretation (6, 47). An extensive work also has been done in the field of aortic valve segmentation for planning transthoracic aortic valve implantation procedure (48, 49, 50).

### Quantification of wall motion abnormalities

One of the widest uses of echocardiography is in the diagnosis and care of heart failure either related to coronary artery disease or other cardiac pathology. Identification and assessment of systolic heart failure relies on identification of wall motion abnormalities. Quantitative assessment of changes in regional wall motion is also important in stress echocardiography to identify patients with prognostically significant coronary disease. Echocardiography allows real-time visualisation of myocardial contractility during stress and by comparing left ventricular wall motion between baseline images and peak or post stress images, it is possible to detect presence of a functionally significant coronary narrowing (51). Typically, contractility is assessed visually by an operator and a meta-analysis of 62 published stress echocardiography studies demonstrated a wide variation in reported sensitivities and specificities for dobutamine stress echocardiography. Sensitivity ranged from 33 to 98%, whilst the specificity ranged from 38 to 97% resulting in average sensitivity and specificity for dobutamine stress echocardiography of 81 and 82%, respectively. Whilst this is comparable to other functional assessments of coronary artery disease, it still means that approximately one in every five patients could potentially be misdiagnosed (52). In order to enhance the accuracy of stress echocardiography, machine learning models have been evaluated as means to identify and quantify inducible wall motion abnormalities (53, 54, 55, 56). In one study, Omar *et al.* used imaging derived models of 3D motion at rest and stress within random forests, support vector machines and a deep learning approach consisting of a convolutional neural network. They found that the convolutional neural network provided the most sensitive model, with a sensitivity of 81.1% in a training dataset compared to expert operator interpretation (55). In another study, an unsupervised learning model was used to detect 12 features for linear discrimination, which could differentiate between patients with obstructive disease and normal responses through use of a new coronary artery disease risk index (54). The majority of studies to date have been on relatively small datasets, without adequate testing validation or have only compared against expert readers rather than outcome. Nevertheless, they show promise that machine learning models may be able to support clinical decision-making for stress echocardiography; one of the most commonly used functional imaging tests for coronary artery disease.

## Conclusion

Although echocardiography is the most accessible imaging modality for the diagnosis of cardiovascular disease, its interpretation remains subjective and operator dependent. In this article, we have highlighted some of the research demonstrating the value of AI, in particular machine learning, in medical imaging and its potential to improve patient care. The inclusion of machine learning models in echocardiography appears very promising, as they are able to accurately identify various echocardiographic features and predict outcomes, without the limitations currently inherent to human interpretation. These technologies therefore have the potential to improve clinical decisions and lead to a reduction in the number of unnecessary investigations, therapies and interventions. Although there are concerns that integration of AI in healthcare settings could replace clinicians, it is more likely that AI will serve as a valuable tool for clinicians, in particular those with less experience, to allow them to treat and diagnose with greater accuracy and confidence. This should have the impact of reducing the opportunity for error and, as such, improve patient care. Whilst the use of machine learning has advanced a great deal over the past decade, the full application is still in its infancy and further research is required to refine and improve its use and implementation in clinical applications.

## Declaration of interest

PL, RU, DM have shareholdings and/or share options in Ultromics Ltd.

## Funding

This research did not receive any specific grant from any funding agency in the public, commercial or not-for-profit sector.
